# Physical Activity versus Sedentary Behavior: Associations with Lipoprotein Particle Subclass Concentrations in Healthy Adults

**DOI:** 10.1371/journal.pone.0085223

**Published:** 2013-12-27

**Authors:** Eivind Aadland, John Roger Andersen, Sigmund Alfred Anderssen, Olav Martin Kvalheim

**Affiliations:** 1 Faculty of Health Studies, Sogn og Fjordane University College, Førde, Norway; 2 Department of Surgery, Førde Central Hospital, Førde, Norway; 3 Department of Sports Medicine, Norwegian School of Sport Sciences, Oslo, Norway; 4 Faculty of Teacher Education and Sports, Sogn og Fjordane University College, Sogndal, Norway; 5 Department of Chemistry, University of Bergen, Bergen, Norway; University of Leicester, United Kingdom

## Abstract

**Background:**

Physical activity (PA) and sedentary behavior (SED) may have independent effects on health and disease. This might be due to PA and SED having distinct effects on lipoprotein metabolism. The aim of this study was to determine associations between lipoprotein subclass particle concentrations (-P) and accelerometer-measured SED and moderate-to-vigorous PA (MVPA) in a sample of healthy adult subjects.

**Methods:**

Lipoprotein subclass particle concentrations were determined by proton nuclear magnetic resonance spectroscopy, whereas SED and MVPA were measured using Agtigraph GT1M and GT3X+ accelerometers. We obtained valid data in 73 subjects (30 men and 43 women, age 40.5 ± 10.6 years; body mass index 24.0 ± 2.8). Multiple regression analysis was used to determine associations (partial correlations) with lipoproteins.

**Results:**

Positive associations were detected between SED and small VLDL-P, large LDL-P and TG (partial r = 0.24 to 0.25, p < .047). Corresponding associations were non-significant for MVPA (partial r = -0.12 to 0.04, p > .355). On the contrary, MVPA was positively associated with large HDL-P, average HDL size, Apo A1 and HDL-cholesterol (partial r = 0.28 to 0.50, p < .027), whereas SED was not (partial r = -0.06 to 0.07, p > .607).

**Conclusion:**

There might be a specific effect of SED versus MVPA on lipoprotein metabolism. However, our results must be interpreted carefully due to possible effect-modification by gender and a low sample size. Thus, our findings should be viewed as preliminary.

## Introduction

There is irrefutable evidence of the effectiveness of regular physical activity (PA) in the primary and secondary prevention of a range of chronic diseases as well as premature death [1-3]. Historically, sedentary behavior (SED) was conceptualized as the lower end of the PA spectrum, as opposed to moderate- to vigorous PA (MVPA), but is now increasingly being viewed as a behavior distinct from PA, defined as any waking behavior characterized by an energy expenditure ≤ 1.5 metabolic equivalents while in a sitting or reclining posture [4]. This is supported by studies that have indicated independent associations between SED and mortality, cardiovascular disease, diabetes type 2 and the metabolic syndrome after adjustment for PA [5-7]. 

The mechanisms behind the possibly distinct effects of SED versus PA are poorly understood. The hypothesis put forth by Hamilton and colleagues [8-11], that SED and PA may have distinct effects on lipid metabolism, as indicated by contrasting effects on lipoprotein lipase activity and transcription, have gained wide attention. However, this hypothesis is not supported by epidemiological studies, as highly inconsistent association patterns between accelerometer-measured SED/MVPA and the standard lipid panel (total cholesterol (TC), low density lipoprotein cholesterol (LDL-C), high density lipoprotein cholesterol (HDL-C) and triacylglycerol (TG)) have been reported across studies in adult to older populations [12-19]. Still, as various subclasses of lipoproteins are quite heterogeneous in size and function, important aspects of the lipid metabolism may be masked by application of the standard lipid panel. This is indicated by findings that PA can favorably alter lipoprotein particle concentrations and apolipoprotein B (Apo B) (a marker for total number of atherogenic lipoprotein particles) independent of LDL-C [20,21]. In a similar fashion, direct measurement of lipoprotein subclass particle concentrations might be critical to reveal distinct impacts of SED versus MVPA on lipoproteins. To the best of our knowledge, associations between accelerometer-measured SED and MVPA and lipoprotein subclass particle concentrations have not been investigated. 

Thus, the aim of the present study was to determine associations between accelerometer-measured SED and MVPA and a larger panel of lipoprotein subclass particle concentrations, determined by proton nuclear magnetic resonance (NMR) spectroscopy, in a sample of healthy adult subjects. Our hypothesis was that SED and MVPA might be associated with distinct lipoprotein patterns in the more finely resolved lipoprotein profiles.

## Methods

### Ethics statement

Written informed consent was obtained from each subject before inclusion in the study. The study conforms to the principles outlined in the Declaration of Helsinki and was approved by The South-East Regional Committee for Medical Research Ethics in Norway.

### Subjects

A healthy group of 78 subjects (45 women and 33 men) was recruited from the general population of a rural county in Western-Norway (all of western European descent) by local media and word of mouth, as part of a study in obese subjects to obtain reference values for NMR spectroscopy-derived blood data. Inclusion criteria were age 18 to 60 years and BMI 18.5 to 29.9. Exclusion criteria were pregnancy, smoking, drug abuse, use of lipid-lowering drugs and established CVD, diabetes type 2 or cancer. 

### Procedures

All assessments (except PA-measurements) were performed on a single time-point between 8 and 13 am. Fasting blood samples were drawn at arrival (between 8 and 9 am). Thereafter, we measured height (to the nearest 0.5 cm), waist circumference (WC) (mean of two measurements to the nearest 0.5 cm) and body weight and fat mass (to the nearest 0.1 kg using bioelectrical impedance analysis (MC 180, Tanita Corp, Tokyo, Japan)) prior to serving breakfast. After breakfast, average diet consumed over the last year was assessed using a validated 180-item food frequency questionnaire [22,23]. Data on diet was analyzed through computer scanning and manually checked for any items added at the Department of Nutrition, The University of Oslo, Norway. At departure, the subjects were instructed how to use the accelerometer for measurement of PA.

Physical activity was measured using an Actigraph GT1M or GT3X+ accelerometer (Actigraph, Fort Walton Beach, FL, USA) and analyzed with the Actigraph software ActiLife v. 5.3. Subjects were instructed to wear the accelerometer over seven consecutive days at all times, except during water activities or while sleeping. A wear-time of ≥ 10 hours/day for ≥ four days was used as the criterion for a valid measure, whereas periods of ≥ 60 minutes (allowing for ≤ 2 minutes of non-zero counts) were defined as non-wear time [24,25]. Physical activity was reported as total PA level (counts/min), minutes/day and percentage of valid wear time in SED, light PA (LPA) and MVPA, using previously applied and established cut points of < 100, 100 – 2019 and ≥ 2020 counts [26], respectively. Percentage time spent at different intensity levels were used in the regressions analyses. For the triaxial accelerometer (GT3X+), only accelerations from the vertical axis were applied. The output from the vertical axis of the GT3X+ and GT1M accelerometers is comparable [27,28]. All measurements were corrected for the self-reported duration of swimming and bicycling (both classified as MVPA [29]) because these modes of activity are poorly captured by the accelerometer and comprised a substantial part of MVPA for many subjects in our sample. 

All blood-samples were drawn after an overnight fast. Lipoprotein subclass particle concentrations were analyzed by proton NMR spectroscopy of native serum samples. The NMR data were measured at 37 °C using a Bruker AVANCE III spectrometer operating at 500.36 MHz using an automated platform [30]. The lipoprotein subclasses were calculated from regression equations with reference measurements of particle size distribution from chromatography as described by Kettunen et al [31]. Originally, six VLDL subclasses, intermediate density lipoprotein (IDL), three LDL subclasses and four HDL subclasses were determined [31], however, these subclasses were combined to define three subclasses (small, medium and large) for VLDL, LDL and HDL particles to be applied in further analyses. The average size of the VLDL, LDL, and HDL particles was calculated by weighting the corresponding subclass diameters with their particle concentrations. Apolipoprotein (Apo) A1 and Apo B were determined from the proton NMR measurements. The samples were analyzed in two batches, where coefficients of variation were 0.3% for lipoprotein size and 1.5 to 7.5% for other variables based on replicate analyses. 

The standard lipid panel (TC, LDL-C, HDL-C and TG) were determined directly by standard laboratory methods (Architect ci 8200, Abbott Diagnostics, Illinois, USA). Typical CV’s reported by the manufacturer were < 2%.

### Statistics

The subject characteristics and data on lipoproteins are presented as the mean values ± standard deviation (SD). In addition, concentrations for large and medium VLDL-P, average VLDL size and TG are presented as medians and interquartile range because the data were skewed. Differences between men and women were tested with the independent samples t-test for most variables, whereas the proportion achieving the guideline PA level was tested using the chi-squared test. The skewed variables were log-transformed for the purpose of this testing.

**Figure 1:**
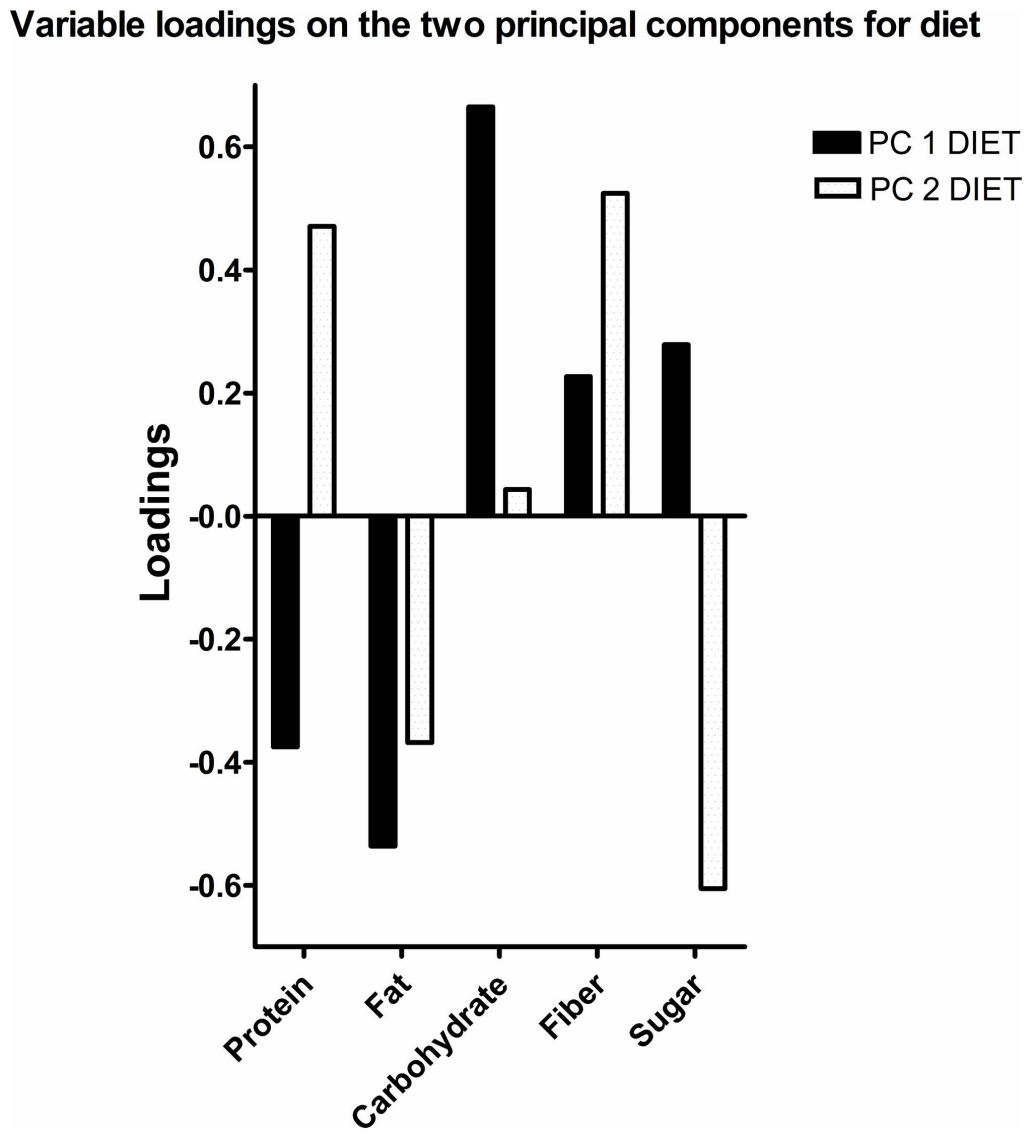
Variable loadings on the two principal components for diet. A higher score on PC 1 DIET indicates that a subject consume (mainly) more carbohydrate and less fat, whereas a higher score on PC 2 DIET indicates that a subject consume (mainly) less sugar and more fiber.

Associations for SED and MVPA versus lipoprotein profile were analyzed in three steps. First, we applied principal component analysis (PCA) to reduce the dimensions in the diet data. Principal component analysis reduces the dimensions in data to a few orthogonal principal components (PCs), were PC 1 has the greatest shared variance between variables and PC 2 the greatest shared residual variance after PC 1 has been accounted for and so forth. Two PCs (PC 1 DIET and PC 2 DIET) were retained (based on five variables: percentage fat, protein, carbohydrate, refined sugar and fiber) having shared variance of 42.9 and 32.2% for PC 1 DIET and PC 2 DIET, respectively (total 75.1%). Loadings on the PCs for are shown in Figure 1. Individual scores on the PCs were extracted for use in further analyses.

Second, independent associations (partial correlations) for SED and MVPA versus each of the 18 lipoprotein variables were analyzed using a multiple regression model adjusting for gender, age, WC, PC 1 DIET and PC 2 DIET. Third, we included the interaction terms SED*gender and MVPA*gender to test for possible effect modification by gender. As several interaction-terms reached a statistically significant level (p < .10), secondary analyses were run for each gender group separately, applying the same model as detailed above. Residuals were normally distributed in all models. 

The PCA were performed using Sirius v. 8.0 (Pattern Recognition Systems, Bergen, Norway). All other analyses were performed using SPSS v. 20 (SPSS Inc., Chicago, USA). A P-value ≤ 0.05 indicated statistically significant findings.

## Results

### Subjects’ characteristics

One male was excluded from the analyses due to very high levels of VLDL-P and TG (z-score > 6) and four subjects (two men and two women) did not provide valid data on PA. Thus, a total of 73 subjects (30 men and 43 women) formed the basis of all results. 

All anthropometric variables differed between men and women (table 1). Males reported a higher energy intake and a lower percentage intake of sugar than women. Women exhibited a somewhat higher PA level than males, however, the only significant difference was detected for the proportion of women (51%) and men (27%) achieving the guideline PA level (≥ 30 min in MVPA/day in bouts of 10 min) (p = .036). Regarding lipoproteins, men and women differed on most variables (table 2). 

**Table 1:** Baseline subject characteristics (mean ± SD).

	**Women**	**Men**	**P** between genders
N	45	33	
Age	40.4 ± 10.6	40.7 ± 10.9	.911
Height (cm)	168 ± 5	180 ± 6	< .001
Weight (kg)	65.6 ± 6.9	81.9 ± 11.6	< .001
WC (cm)	76.3 ± 7.6	88.4 ± 8.4	< .001
BMI (kg/m^2^)	23.2 ± 2.2	25.2 ± 3.2	< .001
Fat mass (kg)	18.4 ± 5.2	15.9 ± 5.5	.045
Lean mass (kg)	47.1 ± 3.3	66.0 ± 7.3	< .001
Fat %	27.7 ± 5.3	19.0 ± 4.8	< .001
PA level (average counts/min)	448 ± 195	388 ± 123	.111
SED (min/day)	554 ± 85	573 ± 85	.346
LPA (min/day)	296 ± 70	282 ± 70	.387
MVPA (min/day)	62 ± 38	53 ± 26	.255
SED (%)	61.1 ± 8.3	63.9 ± 9.0	.169
LPA (%)	32.5 ± 6.7	31.2 ± 7.7	.439
MVPA (%)	6.8 ± 4.2	5.9 ± 3.0	.288
Energy intake (MJ)	9.0 ± 3.2	12.1 ± 2.8	.004
Fat (%)	36.3 ± 5.9	34.2 ± 6.7	.152
Protein (%)	17.8 ± 3.5	18.0 ± 3.9	.611
Carbohydrate (%)	42.2 ± 5.8	43.2 ± 8.7	.521
Sugar (%)	6.0 ± 2.7	5.6 ± 4.3	.015
Fiber (%)	2.6 ± 0.7	2.3 ± 0.6	.595
PC1 DIET (arbitrary unit)	-0.03 ± 1.16	0.04 ± 1.82	.831
PC2 DIET (arbitrary unit)	0.01 ± 1.16	-0.02 ± 1.43	.913

WC = waist circumference; BMI = body mass index; PA = physical activity; SED = sedentary behavior; LPA = light physical activity; MVPA = moderate to vigorous physical activity; PC_1/2_ DIET = principal component number 1/2 for diet

**Table 2:** Lipoprotein particle subclass concentrations, average lipoprotein particle sizes, lipoprotein cholesterol concentrations and apolipoprotein concentrations in women and men.

Variable	Women	Men	**P** between genders
Large VLDL-P (nmol/l)	1.44 ± 1.91 (0.63, 2.25)	3.44 ± 2.87 (2.92, 3.85)	.001
Medium VLDL-P	7.73 ± 6.04 (5.67, 6.85)	13.66 ± 6.84 (12.45, 10.15)	< .001
Small VLDL-P	51.3 ± 18.4	69.2 ± 19.0	< .001
Large LDL-P (nmol/l)	222 ± 67	261 ± 66	.012
Medium LDL-P	107 ± 34	129 ± 33	.005
Small LDL-P	122 ± 36	144 ± 36	.012
Large HDL-P (μmol/l)	1.78 ± 0.54	1.09 ± 0.37	< .001
Medium HDL-P	1.87 ± 0.24	1.74 ± 0.28	.021
Small HDL-P	4.22 ± 0.36	4.41 ± 0.33	.020
VLDL-size (nm)	35.11 ± 1.17 (34.88, 1.42)	36.09 ± 1.22 (36.06, 2.13)	< .001
LDL-size	23.64 ± 0.16	23.64 ± 0.13	.993
HDL size	10.12 ± 0.25	9.80 ± 0.17	< .001
Apo A1 (g/l)	1.72 ± 0.19	1.56 ± 0.19	< .001
Apo B	0.81 ± 0.22	0.97 ± 0.22	.002
TC (mmol/l)	5.08 ± 1.06	5.36 ± 0.99	.241
LDL-C	3.07 ± 1.02	3.61 ± 0.88	.017
HDL-C	1.60 ± 0.29	1.27 ± 0.26	< .001
TG	0.78 ± 0.34 (0.66, 0.38)	1.11 ± 0.42 (1.03, 0.59)	< .001

Values are means ± SD (median, interquartile range). VLDL-P = very low density lipoprotein particle concentration; LDL-P = low density lipoprotein particle concentration; HDL-P = high density lipoprotein particle concentration; Apo = apolipoprotein; TC = total cholesterol; LDL-C = low density lipoprotein cholesterol; HDL-C = high density lipoprotein cholesterol; TG = triacylglycerol

### Association for SED and MVPA versus lipoproteins

Ten men (33%) and 19 women (44%) reported performing a mean of 19 ± 15 (minimum 5 to maximum 47) and 11 ± 7 (2 to 25) minutes of swimming plus bicycling per day, respectively. This added on average 44 and 22% to their accelerometer-determined minutes of MVPA/day, which in the total group amounted to an 11% increased level of MVPA. 

**Figure 2:**
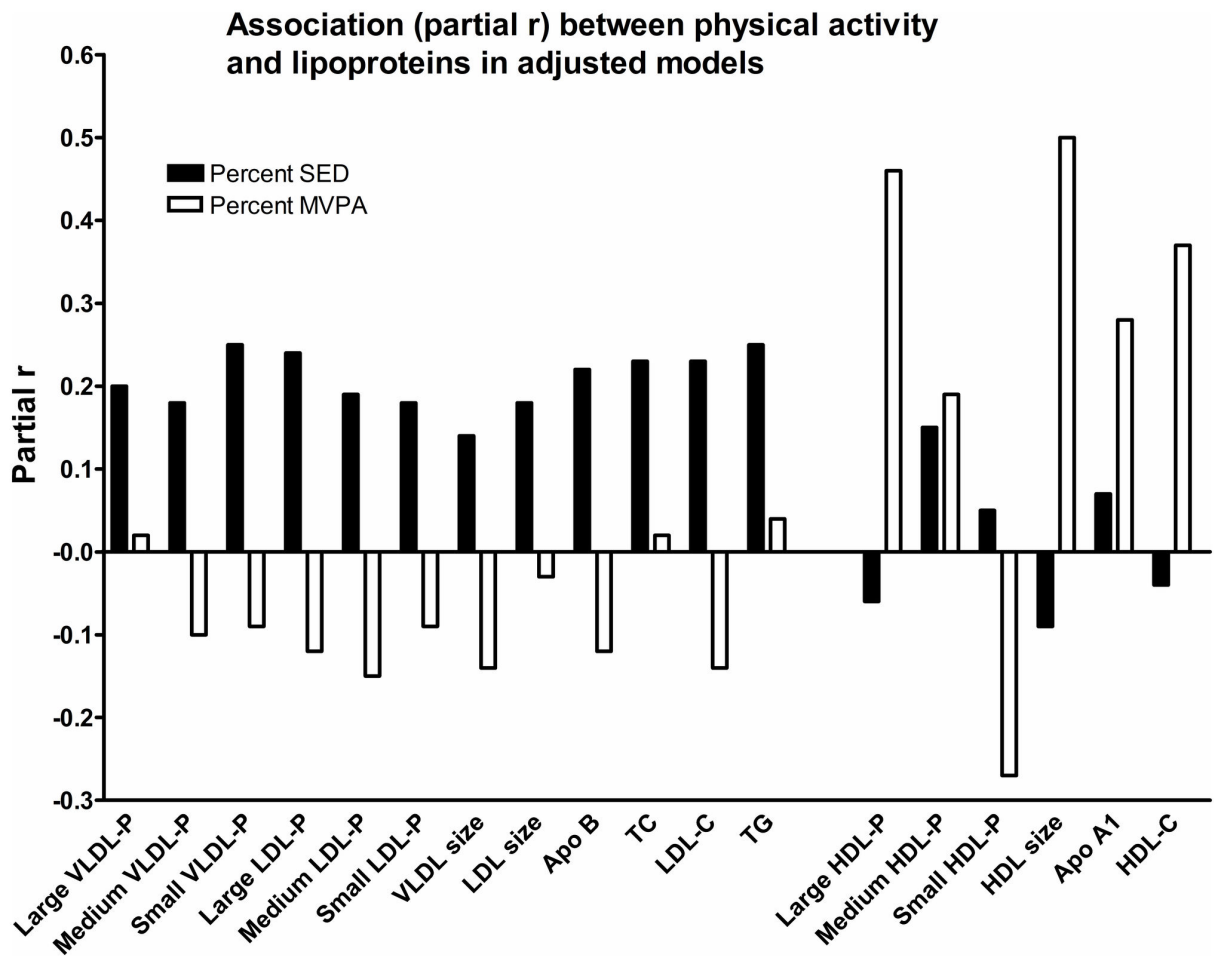
Associations between SED/MVPA and lipoproteins. Pattern of associations (partial r) for SED versus MVPA with lipoprotein subclass particle concentrations (associations are tested with each lipoprotein particle separately based on a full model adjusted for gender, age, WC and diet). Partial rs ≥ 0.24 are statistically significant. (VLDL-P = very low density lipoprotein particle concentration; LDL-P = low density lipoprotein particle concentration; HDL-P = high density lipoprotein particle concentration; Apo = apolipoprotein; TC = total cholesterol; LDL-C = low density lipoprotein cholesterol; HDL-C = high density lipoprotein cholesterol; TG = triacylglycerol).

The inter-correlation between SED and MVPA were r = -0.27 (p = .022). The pattern of relationships between SED and MVPA versus lipoproteins is shown in Figure 2. Generally, somewhat stronger associations with VLDL-P, LDL-P, Apo B and TG were detected for SED compared to MVPA, whereas a converse pattern was seen for HDL and Apo A1. Specifically, relationships between MVPA and large HDL-P and average HDL size were quite strong (partial r = 0.46 and 0.50, respectively, p < .001), whereas the corresponding relationships for SED were non-significant (p > .494).

**Figure 3:**
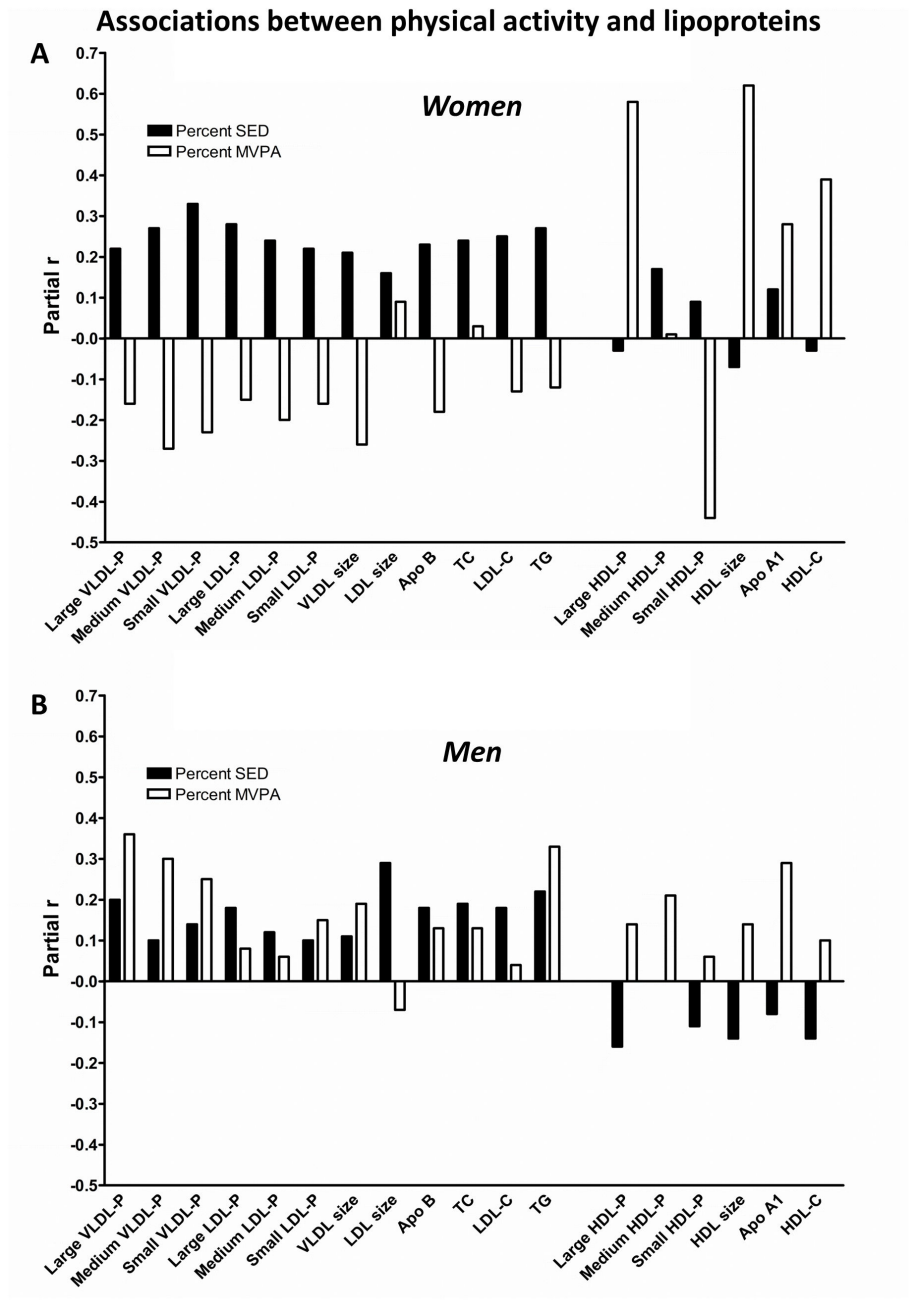
Associations between SED/MVPA and lipoproteins in each gender group. Pattern of associations (partial r) for SED versus MVPA with lipoproteins subclass particle concentrations (associations are tested with each lipoprotein particle separately based on a full model adjusted for gender, age, WC and diet) in **a**) women and **b**) men. Partial rs ≥ 0.33 are statistically significant for women; no associations were statistically significant in men. (VLDL-P = very low density lipoprotein particle concentration; LDL-P = low density lipoprotein particle concentration; HDL-P = high density lipoprotein particle concentration; Apo = apolipoprotein; TC = total cholesterol; LDL-C = low density lipoprotein cholesterol; HDL-C = high density lipoprotein cholesterol; TG = triacylglycerol).

Gender-interactions (gender*MVPA and gender*SED) were included in all regression models to test for possible effect-modification by gender. Generally, associations between SED/MVPA and lipoproteins were stronger for women than for men, although no interaction-terms reached a statistically significant level for gender*SED (p > .284). The gender*MVPA term was significant for all VLDL-P subclasses, TG and small HDL-P (p < .097). Due to these findings, secondary analyses were run for men and women separately, shown in Figure 3. While the pattern of associations for SED and MVPA versus lipoproteins among women was quite similar to findings in the total group, this pattern was not found in the male group (no significant associations between either SED or MVPA and lipoproteins were detected). 

## Discussion

The main finding of this study was that there might be a specific effect of SED versus MVPA on lipoprotein metabolism. Generally, the strongest associations for SED were detected with VLDL, LDL, Apo B, TC and TG, whereas the strongest associations for MVPA were detected with HDL and Apo A1. However, the results indicated that the effects of MVPA might be gender-specific, which means that our results must be interpreted carefully due to the small sample size included.

Sedentary behavior has gained wide attention the last decade as a risk-factor for mortality, cardiovascular disease, diabetes type 2 and the metabolic syndrome, possibly independent of MVPA [5-7]. The distinction between SED and PA has been supported by the work by Hamilton and colleagues [8-11] who hypothesized that the sedentary physiology might be different from the exercise physiology in terms of cellular adaptations affected, specifically regarding lipoprotein metabolism. The present study might support this notion, as the patterns of associations differed somewhat between MVPA and SED. The strongest relationships with MVPA were found with increase in HDL and Apo A1, whereas SED was related to increase in VLDL, LDL, Apo B, TC and TG. However, it does not seem to be any agreement in the literature regarding associations between SED/MVPA and lipoprotein-cholesterol and TG, neither in terms of strength of the relationships nor any specific impact of SED versus MVPA [12,13,15,18,19]. Both SED and MVPA have been associated with TC, LDL-C, HDL-C and TG with SED displaying the strongest relationships [15]; both measures have been associated with TG and HDL-C with MVPA displaying the strongest relationship [18]; SED and MVPA have been significantly related to TG, but not to HDL-C [13]; MVPA have been significantly related to TC and TG, but not HDL-C, while SED did not relate to any variable [12]; neither measure have been associated with TG nor HDL-C [19]. 

We have few suggestions regarding what might explain such differences across studies, besides an inherent variation in estimates over study settings and populations. Previous studies have relied on the standard lipid panel, whereas we applied NMR-analysis to measure lipoprotein subclass particle concentrations. This procedure clearly provides a more detailed picture of the lipoprotein profile compared to application of the standard lipid panel. For example, HDL-particles are highly heterogeneous [32] as also indicated by our findings when comparing associations with SED/MVPA for large versus medium and small HDL-P. For women, association between MVPA and large HDL-P (partial r = 0.58, p < .001) and average HDL size (partial r = 0.62, p < .001) was quite strong, whereas a weaker association was detected for HDL-C (partial r = 0.39, p = .016). Thus, reliance on the standard lipid panel might be one reason why systematic patterns are not revealed.

Another factor explaining some variability might be variation in accelerometer-data handling. Because SED may be easily confused with non-wear time, minutes of SED are likely to be influenced by valid wear-time. Thus, analyses might be adjusted for wear-time [12] or be expressed relative to total wear-time (as in the present study). Percent SED has previously been shown to be superior to minutes of SED in relation to metabolic risk [17]. In the present study, expression of SED and MVPA as actual or relative time did not change any findings. Furthermore, certain modes of PA (for example swimming, bicycling and activities demanding upper body work) are poorly captured by accelerometry, thus, variation in such activities across populations and individuals might disturb study findings. We corrected estimates of SED and MVPA for self-reported duration of swimming and cycling, which are not captured by the accelerometer. However, although time on these activities added 11% to accelerometer-determined MVPA, associations with lipoproteins were virtually unchanged from uncorrected estimates.

Effect-modification by gender might also be a factor affecting study results, at least, it challenge the interpretation of our findings. Whereas relationships between MVPA and VLDL, LDL, Apo B, TC and TG were more or less negative in women, they tended to be positive in men, although no variables reached a statistically significant level in the male group. Yet, when compiling data for men and women, the opposite relationships partly cancelled each other out, leading to quite weak relationships between MVPA and these variables (especially with VLDL-P and TG for which the MVPA*gender-interaction reached a statistically significant level). In the female group, MVPA and SED were quite similarly related to VLDL, LDL, Apo B, TC and TG. Still, the relatively strong relationships between MVPA (as opposed to SED) and HDL and Apo A1 remained, which suggest that HDL levels might be more affected by MVPA compared to SED. 

It is well known that lipid metabolism [33] and lipoprotein particle concentrations [34] differ by gender. Studies have also reported that SED might be somewhat stronger associated with metabolic risk in women compared to men [6], but this might be an issue of measurement [35]. We have no obvious explanation for the gender-specific effects of MVPA found in the present study. Although the full model was adjusted for age, waist circumference and diet, residual confounding due to low measurement-precision for diet or confounding by unknown factors could explain the findings. Secondly, the significant interaction-terms could be caused by unstable associations owing to the small sample size, however, quite large interaction-effects is necessary to reach a statistically significant level in small samples. Of the previous studies that have compared accelerometer-determined SED and MVPA in relation to lipoproteins [12,13,15,18,19], only one study [12] reported testing of effect-modification by gender (found to be non-significant). 

A body size above normal weight (BMI > 25) is associated with increased risk of CVD and mortality [36]. Still, lipoprotein-cholesterol profiles seem to be adversely affected by increased fatness also in normal-weight subjects [36]. In line with this, WC was significantly associated with several lipoprotein subclass concentrations in our sample of normal- to overweight men and women (results not shown). However, our results indicate that an active lifestyle can mitigate the unhealthy effect of fatness on metabolic health even in an active healthy population, showing the potential for an active lifestyle in primary prevention of CVD. This is consistent with evidence that PA and high aerobic fitness is protective for CVD and mortality, independent of obesity [37,38], although PA might not affect standard lipid risk factors for CVD to a large extent [37]. 

### Strengths and weaknesses

The present study has two main strengths. First, lipoprotein subclass particle concentrations were measured using NMR-analysis, which clearly provide a more detailed picture of the lipoprotein profile compared to application of the standard lipid panel. Therefore, we believe our data was well suited to answer the research question being posed. Second, PA was measured objectively using an accelerometer. Due to the highly inconsistent relationships found between self-reported and objectively measured PA [39], and also considering that stronger associations has been found with metabolic risk for objectively measured versus self-reported SED and MVPA [14,15], we believe objective measurement of SED and MVPA was of critical importance in the present study. 

The main limitation of the present study was the small sample size, especially considering that we identified gender-specific associations and performed secondary analyses in each gender-group separately. Thus, the study findings might be unstable and must be interpreted carefully. Our results should be viewed as preliminary, until examined in studies with greater samples size. Second, we determined associations for SED and MVPA with each single lipoprotein variable in the total sample as well as in each gender group, without correction of our ɑ-level. This may have inflated our type 1 error rate. However, we report on the pattern of associations and did not aim to detect associations with specific lipoproteins. Third, diet was measured using a food-frequency questionnaire being subject to many well-known limitations (40,41). Finally, the study is cross-sectional in nature, therefore, no causality could be inferred from our findings.

Future research should seek to test and possibly verify the present study-findings in a larger sample of men and women. Specifically, studies including both gender groups are warranted as there are certain gender differences in the lipid metabolism [33,34], and since associations between SED/MVPA and lipoproteins might be moderated by gender, as shown in the present study. The biological mechanisms behind the possibly different effects of SED and PA on lipoproteins should also be explored. 

## Conclusions

We conclude that there might be a specific effect of SED versus MVPA on lipoprotein metabolism. Generally, SED was associated with increased VLDL, LDL, Apo B, TC and TG, whereas MVPA were associated with increased HDL and Apo A1. However, because the results indicated that the associations with MVPA might be gender-specific and because we included a low sample size, our results must be interpreted carefully. Future studies are encouraged to determine associations between accelerometer-determined SED/PA and lipoprotein subclass particles in larger samples of men and women. Until then, our results should be viewed as preliminary.
